# Bile acid signaling mediates gut microbiota regulation of host lipid metabolism

**DOI:** 10.3389/fmicb.2025.1696213

**Published:** 2026-01-13

**Authors:** Mengkuan Liu, Cuimei He, Xinyu Lv, Wanru Zheng, Jie Yin, Yixing Li, Jie Ma

**Affiliations:** 1 Guangxi Key Laboratory of Animal Breeding, Disease Control and Prevention, College of Animal Science and Technology, Guangxi University, Nanning, China; 2 College of Animal Science and Technology, Hunan Agricultural University, Changsha, China

**Keywords:** gut microbiota, bile acids, lipid metabolism, signal pathway, enterohepatic circulation

## Abstract

Although advancements in the study of gut microbiota have clarified the close association between gut microbiota and the host's metabolism, the underlying mechanism remains unclear. Bile acids are important components of the host's metabolism and are deeply influenced by gut microbiota. In recent years, significant progress has been made in the study of how gut microbiota regulate their host's lipid metabolism through bile acids. This review summarizes the enterohepatic circulation of bile acids, the interaction between gut microbiota and bile acids, and the role of gut microbiota-bile acid signaling in the regulation of lipid metabolism. Finally, this review examines the effects of bile acid metabolism disorders on obesity. This review aims to provide a novel perspective for understanding the relationship between gut microbiota and the host's lipid metabolism.

## 1 Introduction

The gut microbiota is a complex, diverse, and constantly changing ecosystem comprising bacteria, fungi, viruses, archaea, and protozoa (Fang et al., 2021). Recently, increasing evidence has shown that gut microbiota played a crucial role in host metabolism, particularly in the regulation of lipid metabolism (Schoeler and Caesar, 2019). Studies have also shown that high-fat diet shift the gut microbiota toward a higher Firmicutes-to-Bacteroidetes ratio, thereby increasing energy acquisition and promoting obesity, while a low-fat diet reversed this phenomenon (Tong et al., 2021). This suggests that the gut microbiota was an important factor that regulated the host's lipid metabolism and deposition. The gut microbiota usually play a regulatory role through their metabolites, including short-chain fatty acids (SCFAs), amino acids, bile acids, polyamines, and lipopolysaccharide (LPS; Du et al., 2023). Importantly, bile acids were signaling molecules and metabolic integrators that can activate nuclear receptors, including farnesoid X receptor, pregnane X receptor, vitamin D receptor, and G protein-coupled bile acid receptor; therefore, they play important roles in regulating lipid metabolism and inflammation (Li and Chiang, 2014). Bile acids also participate in lipid metabolism through the enterohepatic circulation, and their metabolism is related to gut microbiota (Xu and Gui, 2024). Before reaching the ileum, bile acids undergo a series of biotransformation processes induced by intestinal microbiota, including dehydrogenation reactions, dissociation effects, oxidation of hydroxyl groups at C3, C7, and C12 positions, and desulfation reactions. These transformation processes were closely regulated by the intestinal microbiota (Jia et al., 2019). Additionally, gut microbiota modify bile acids, leading to changes in receptor signaling and microbial population, which may affect the host's metabolic status and regulate lipid metabolism (Agus et al., 2021). This review aims to examine the latest advances in studies on the underlying mechanisms through which intestinal microbiota regulate host lipid metabolism through bile acids and to provide insights for related research fields.

## 2 Bile acid enterohepatic circulation

**Figure 1:**
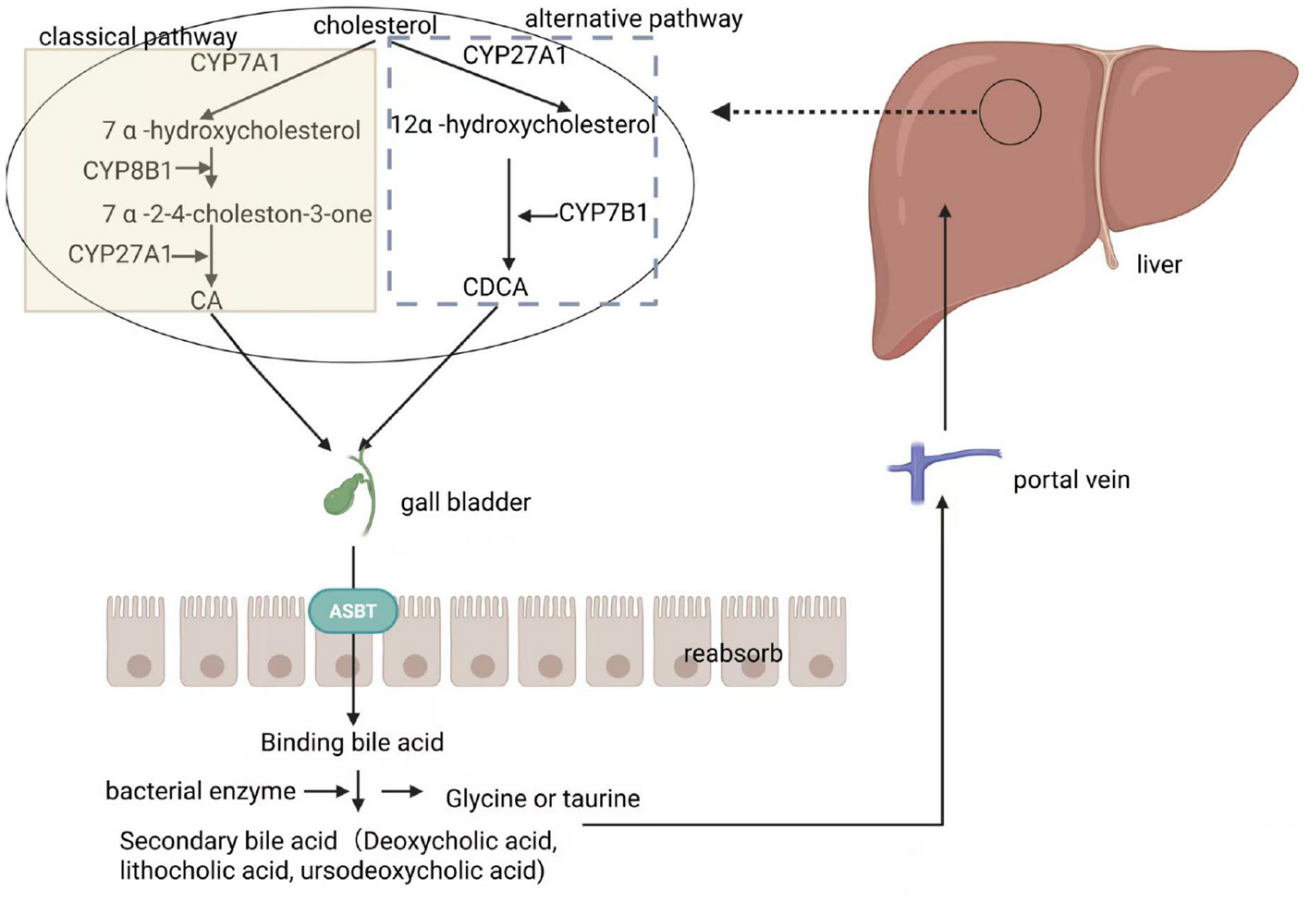
Enteric-hepatic circulation of bile acids.

Bile acid enterohepatic circulation ensures effective recovery and reuse of bile acids. Bile acids are organic acids synthesized by the liver and secreted into bile. These compounds play vital roles in the digestion, absorption, and metabolism of lipids (Figure 1). There are two pathways for bile acid synthesis: The primary pathway, accounting for over 75% of bile acid synthesis, is also known as the classical or neutral pathway. This process is mediated by CYP7A1 (cholesterol 7α-hydroxylase), its rate-limiting enzyme, which catalyzes cholesterol 7α-hydroxylation (Yu and Kiat, 2024). Subsequently, CYP8B1 (Sterol 12α-hydroxylase) participates in catalyzing the oxidative cleavage of the cholesterol side chain. The oxidized cholesterol products undergo further hydroxylation catalyzed by CYP7B1 (Oxysterol 7α-hydroxylase), ultimately producing primary bile acids, specifically cholic acid (CA; Chang, 2025). The alternative pathway, also known as the acidic pathway, is initiated by mitochondrial cytochrome P450 enzyme 27-hydroxylase (Cholesterol 27α-hydroxylase, CYP27A1; Chiang, 2013). This enzyme catalyzes the oxidation of sterol side chains, followed by the cleavage of three-carbon units in the lipoxygenase complex. This process leads to the formation of C-24 bile acids, which are ultimately transported to the liver, where oxidative metabolites were converted into chenodeoxycholic acid (CDCA; Gao, 2023). In this process, hydroxylation at the C-12 position is essential for bile acid formation. The classical pathway, involving CYP8B1 (12α-hydroxylase), produces both 12-hydroxylated and non-12-hydroxylated bile acids. In contrast, the alternative pathway lacks CYP8B1 (12α-hydroxylase) and exclusively generates non-12-hydroxylated bile acids (Wang Y. et al., 2024). Bile acids are then secreted into the intestine through the bile ducts to participate in the digestion and absorption of lipids.

Primary bile acids are converted into secondary bile acids by gut microbiota in the intestines (Liu et al., 2023). Several secondary bile acids are amphiphilic bile acids, which are more suitable for the dissolution and excretion of cholesterol and fat in the body. First, enzymes from gut bacteria induce a deconjugation reaction in which bile acid molecules were separated from the glycine or taurine molecules to which they are bound. This step facilitates the subsequent transformation process, leading to further modifications (Pushpass et al., 2022). Subsequently, under the catalysis of bacterial enzymes, bile acid molecules begin to undergo the dehydroxylation reaction, leading to the synthesis of a series of secondary bile acids with different chemical structures and properties, including deoxycholic acid, lithocholic acid, and ursodeoxycholic acid, that play important roles in metabolic processes in the human body (Wang and Zhao, 2024). Finally, secondary bile acids are reabsorbed in the intestines and return to the liver through the portal vein, allowing them to repeatedly participated in the synthesis and secretion of bile (Kumar Singh et al., 2019).

This figure shows the metabolic cycle of bile acids in the body, covering the complete process from cholesterol synthesis of primary bile acids to intestinal transformation, reabsorption, and enterohepatic circulation.

## 3 Relationship between gut microbiota and bile acid metabolism

### 3.1 Gut microbes are involved in bile acid metabolism

As an important metabolite in the host, the formation and metabolism of bile acids are significantly affected by the gut microbiota. While changes in the gut microbiota directly affect the metabolism of bile acids, *Bifidobacterium* and *Lactobacillus* (e.g., *Lactobacillus rhamnosus, Lactobacillus acidophilus*, and *Lactobacillus reuteri*) can produce bile salt hydrolytic enzyme (BSH), which participates in the deconjugation of bile acids, converting conjugated primary bile acids into free bile acids. The increase in the ratio of *Bacteroides, Proteus, Firmicutes, Bacteroides*, and *Actinomycetes* can increase secondary bile acids, among which *Actinomycetes* can promote the excretion and synthesis of bile acids (Table 1). The gut microbiota affects the metabolic transformation of bile acids through a variety of enzymatic reactions and signal regulation mechanisms. BSH and 7α-dehydroxylase produced by the gut microbiota are key enzymes in the uncoupling reaction of bile acid metabolism. After BSH and dehydrogenation, primary bile acids are converted into secondary bile acids, such as LCA, DCA, UDCA, and ωMCA (Foley et al., 2021; Ridlon et al., 2016). BSH is mainly produced by intestinal flora, such as lactic acid bacteria and *Bifidobacterium*, and is highly conservatively expressed in these organisms (Collins et al., 2023). Bile salts produced by the gut microbiota are mainly involved in the process of dissociating conjugated bile acids into free bile acids in the host. Subsequently, gut microbes convert CA and CDCA into DCA and LCA through 7α-dehydroxylase and convert UDCA to LCA through 7β-dehydroxylase (Foley et al., 2021). Moreover, studies have found that gut microbes can further convert α-MCA and β-MCA into murine deoxycholic acid (ωMCA) in mice (Ridlon et al., 2016). The increase of BSH activity enhanced the content of free bile acids, triggering the FXR-FGF15/19 negative feedback regulatory system for bile acids, reducing bile acid synthesis, and causing cholesterol to become supersaturated when gut microbiota is imbalanced (Fan et al., 2024). If bile acids cannot be effectively dissolved, they may precipitate to form stones. Importantly, gut bacteria, especially *Lactobacillus* and *Bifidobacterium*, can eliminate cholesterol by ingesting cholesterol or forming co-precipitates through assimilation (Horáoková et al., 2018). Furthermore, some bacteria convert cholesterol into insoluble prostaglandins, allowing them to be excreted through feces. The FXR-FGF15-CYP7A1 pathway also plays an important role in regulating the synthesis of primary bile acids by gut microbiota (Sayin et al., 2013).

**Table 1:** Gut microbiota altered composition of bile acid.

**Gut microbiota variation**	**Composition of bile acid**	**References**
*Bifidobacterium*↑	GDCA and TDCA↑	Chen et al., 2023
*Lactobacillus rhamnosus↑*	Liver chenodeoxycholic acid level↑	Liu Y. et al., 2020
*Lactobacillus acidophilus↑*	Cholic acid and chenodeoxycholic acid↓	Wu et al., 2024
Proportion of *Firmicutes* and *Bacteroides*↑	CDCA concentration↑, LCA, DCA, UDCA, and HDCA concentration↓	Li et al., 2017
*Actinomycetes*↑	The excretion of bile acids and bile acid synthesis↑ CA concentration↑	Degirolamo et al., 2014
*Bacteroides* and *Proteus*↓	Concentrations of GDCA and TUDCA↓	Degirolamo et al., 2014; Qi et al., 2019
*Lactobacillus acidophilus↑*	Concentrations of CDCA and DCA↑	Tawulie et al., 2023
*Lactobacillus reuteri↑*	Secondary bile acid↑	Collins et al., 2023
*Clostridium↑*	Concentration of β-MCA, TCA, tauroursodeoxycholic acid (TUDCA), and UDCA in feces↑	Łukawska and Mulak, 2022

On the one hand, the BSH produced by *Lactobacillus* reduces the reabsorption of bile acids in the small intestine. This metabolic process increases the excretion of bile acids in feces, which in turn leads to more cholesterol being converted into bile acids, thereby reducing serum cholesterol levels (Wang and Xue, 2020). On the other hand, free bile acids produced during the BSH decoupling process are difficult to digest and absorb in the intestines, resulting in an increase in the amount of free bile acids eliminated in feces (Pereira et al., 2003). Consequently, more cholesterol in the liver is converted into bile acids through the bile acid-FXR feedback mechanism (Choi et al., 2015), thereby reducing the overall cholesterol content. Some traditional Chinese medicine extracts that significantly increase the number of *Lactobacillus* in the intestines of poultry and livestock have been found to promote the proliferation of other beneficial bacteria in the intestines of Gallus gallus domesticus and accelerate the excretion and absorption of bile acids (Jiang et al., 2017; Wang Z. et al., 2024). Since *Lactobacillus* secrete acidic substances, these substances reduced the pH of the intestines and the solubility of bile acids, thereby inhibiting the absorption of bile acids in the intestines (Vivekanandan et al., 2024; Hong et al., 2015) and increasing the concentration of fecal bile acids. Furthermore, a recent study showed that adding *Lactobacillus plantarum* increased the fecal bile acid excretion of patients with hyperlipidemia by 15% and reduced the serum levels of low-density lipoprotein cholesterol (LDL-C) by 10% (Meng, 2023). Moreover, studies have found that in mice, antioxidants (tempol) preferentially reduced the abundance of *Lactobacillus* and BSH activity and increased the content of intestinal taurine-β-mouse bile acid (T-β-MCA; Li et al., 2013; Gonzalez et al., 2016). *Bifidobacteria* also affected bile acid metabolism by producing BSH. For example, the hydrolase BSH produced by *Bifidobacteria* rapidly hydrolyzes T-β-MCA into β-MCA (Tanaka et al., 1999) and reduced the inhibitory effect on the FXR signaling pathway (Wang and Xue, 2020). The number of Bifidobacteria in the gut of mice was increased by resistant starch and natural extracts [such as Salvia miltiorrhiza and resveratrol), and then decompose primary bile acids (deoxycholic acid (DCA) and lithocholic acid (LCA)] into secondary bile acids [such as glycodeoxycholic acid (GDCA) and taurodeoxycholic acid (TDCA)] through BSH (Li et al., 2024, 2021; Liu et al., 2024). However, if the levels of beneficial bacteria such as *Bifidobacteria* was decreased, a large amount of conjugated bile acids will be reabsorbed by the liver, and the enzymatic reaction for bile acid synthesis will be inhibited, resulting in a decrease in bile acid secretion (Qiu et al., 2025).

Additionally, other bacterial genera are also involved in regulating bile acid metabolism. For example, some strains of the genus *Clostridium* (such as *Clostridium*) convert lithocholic acid (LCA) to 3-oxo-LCA, which had antibacterial activity, through the action of 3β-hydroxysteroid dehydrogenase, thereby inhibiting the proliferation of intestinal pathogens (Cai et al., 2022). Another study showed that providing mice with different types of mixed live bacteria combinations (such as *Bifidobacterium breve, Bifidobacterium longum, Lactobacillus anaerobius, Lactobacillus plantarum, Lactobacillus helveticus*, pasteurized milk yeast, and *heat-sensitive Streptococcus* formed after high-temperature fermentation) promoted the excretion of more free bile acids (Winston and Theriot, 2020). It was further found that the production of new bile acids was stimulated by the inhibition of the FXR-FGF15 pathway from the colon to the stomach (Pathak et al., 2018). The above studies indicate that gut microbes can regulate bile acid metabolism by producing BSH.

### 3.2 Effect of bile acids on intestinal microbial composition

The change of bile acid also has a great influence on the composition of the gut microbiota. Studies have shown that bile acids inhibit the growth of harmful bacteria, such as *Escherichia coli, Streptococcus*, and *Salmonella*, by destroying bacterial cell membranes or regulating intestinal pH, thereby maintaining the homeostasis of the gut microbiota (Ridlon et al., 2016). Secondary bile acids such as deoxycholic acid increased the proportion of *Bacteroidetes* and reduce the proportion of *Firmicutes, Bacteroidetes*, and *Lactobacillus* through FXR (Qian et al., 2018; Xu et al., 2021). Furthermore, chenodeoxycholic acid inhibited the proliferation of harmful bacteria in the gut, such as *Clostridium, Toona*, and *Erysipelothrix* (Islam et al., 2011), indicating that secondary bile acids reshaped the composition of the intestinal microflora and were closely related to intestinal inflammation and diseases. Interestingly, primary bile acids, such as taurocholic acid, increased the microbial diversity of neonatal mice and significantly reduce the abundance of *Escherichia coli*, while ursodeoxycholic acid did not cause this change (van Best et al., 2020). Further research also found that changed in the concentration and composition of bile acids affect the diversity of gut microorganisms; 7α-dehydroxyl bacteria can rapidly dissolve the cell membranes of bacteria and cause the outflow of bacterial contents; however, their growth was inhibited with the increase in BA concentration, thus preventing ecological imbalance and the release of inflammatory markers (Bustos et al., 2018). However, the decrease in BA concentration was conducive to the growth of Gram-negative bacteria, which induced the release of inflammatory markers and increased the inflammatory response of the liver (Wang Y. et al., 2023). Additionally, the increase in bile acid concentration led to a decrease in the proportion of some beneficial bacteria such as *Lactobacillus* and *Bifidobacteria*, while the proportions of some potentially harmful bacteria were increased (Li, 2023). However, there is no complete pathway defining how bile acids regulate gut microbiota.

## 4 Gut microbiota regulates host lipid metabolism through bile acid signaling

**Figure 2:**
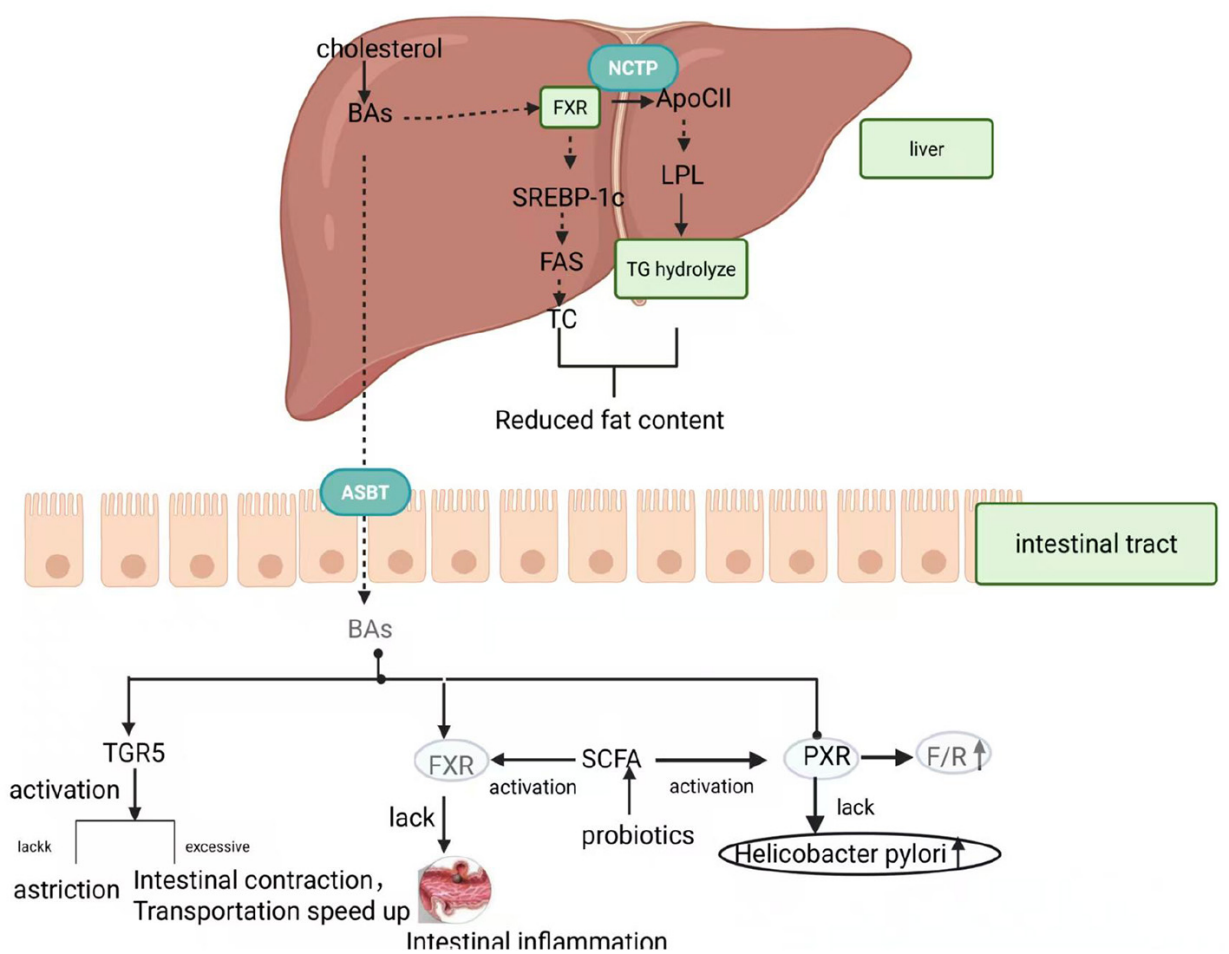
Function of bile acid signaling.

Bile acids participate in regulating substance metabolism and maintain the homeostasis of the host's internal environment by activating several types of receptors. Currently known bile acid receptors were divided into two categories: the first category comprises nuclear receptors, including FXR, PXR, VDR and CAR, while the other category comprises membrane receptors, mainly TGR5 and S1PR2 (Ticho et al., 2019). Bile acid signaling, as a key mode of communication between the gut microbiota and the host, has attracted much attention from researchers in recent years (Figure 2). However, how gut microbes regulate lipid metabolism through bile acid signaling requires further detailed study.

This figure shows the mechanism by which bile acids regulate lipid metabolism, intestinal function, and microbiota interaction through the liver-gut axis.

### 4.1 Bile acids and farnesoid X receptor (FXR)

The FXR receptor is a ligand-regulated nuclear receptor, with bile acids being one of its ligands. Additionally, CA, DCA, and LCA act as agonists of FXR, while UDCA, TαMCA, and Tβ-MCA serve as its natural antagonists (Wahlström et al., 2016). FXR is expressed in multiple tissues, including the liver, intestines, kidneys, and adrenal glands, with the highest expression being in the liver and intestines. The main receptor of FXR is bile acid, and its activation is important for the regulation of bile acid metabolism (Teodoro et al., 2011). Studies had shown that the activation of FXR can promote the diversity and stability of the gut microbiome, thereby improving the host's gut health. For instance, in FXR-deficient mice, the diversity of the gut microbiome is significantly reduced, accompanied by challenges such as intestinal inflammation (Gadaleta et al., 2020; Li et al., 2021). Early colon cell studies have revealed that FXR-deficient mice are more prone to developing severe intestinal inflammation compared to wild-type mice, suggesting that intestinal FXR can alleviate intestinal inflammation (Ma et al., 2025). When treated with FXR agonists, these problems can be significantly improved, enhancing the diversity and stability of the gut microbiome (Wang H. et al., 2024). The activation of FXR can influence the host's immune response by modulating the composition of the gut microbiome. Some studies have also shown that FXR suppresses the occurrence and development of intestinal inflammation (Paik et al., 2022), and this may be related to FXR's regulation of anti-inflammatory substances produced by the gut microbiome. Additionally, FXR can promote the integrity of the intestinal barrier, thereby preventing harmful substances from entering the bloodstream and protecting the host from pathogenic and toxic invasions (Zhuang et al., 2023). Furthermore, FXR also influenced the host's energy metabolism and insulin resistance by regulating the metabolism of the gut microbiome. For example, studies have shown that the activation of FXR promotes the production of short-chain fatty acids by the gut microbiome, which were then absorbed and utilized by the host as an energy source, leading to improvement in insulin resistance in the host (Han et al., 2021).

Further research on bile acids had led to the discovery of the important relationship between them and their receptor, FXR, and how they affect lipid metabolism. Experimental evidence has shown that knocking out the FXR gene in mice significantly increased the total triglyceride content in the blood (Moschetta et al., 2004). This also affected Sterol Regulatory Element-Binding Protein (SREBP), a molecular switch belonging to a group of proteins that integrated into cells and served as an important regulatory factor for fat synthesis (Debose-Boyd and Ye, 2018). This is because endogenous bile acid-activated FXR mediates the downregulation of SREBP-1c expression and decreases the expression of fatty acid synthase (FAS) in a SHP-dependent manner, thereby inhibiting liver fat production (Watanabe et al., 2004). Another important transcriptional factor, carbohydrate response element binding protein (ChREBP), had been shown to promote carbohydrate conversion to lipids and inhibit lipid synthesis through bile acid activation of FXR to downregulate ChREBP expression (Duran-Sandoval et al., 2005). Furthermore, peroxisome proliferator-activated receptor alpha (PPAR-α) plays a role in regulating lipid metabolism and fatty acid oxidation; its activation by FXR leads to increased fatty acid oxidation metabolism and reduced fat deposition (Li and Chiang, 2009).

Membrane receptor SRB1 was a protein responsible for removing high-density lipoprotein cholesterol (HDL-C) from the blood and was regulated by FXR. The activation of hepatic FXR leads to reduced phosphorylation of p-JNK, subsequently stimulating the expression of Hepatocyte Nuclear Factor 4 alpha (HNF4α) and SRB1, further enhancing the clearance capacity of HDL-C (Zhang et al., 2010). On the other hand, the absence of FXR demonstrated that elevated levels of high-density lipoprotein (HDL) cholesterol in plasma are regulated by SRB1 (Lambert et al., 2003). ApoCII was another target gene of FXR whose increased expression promoted the production of lipoprotein lipase (LPL), leading to an increase in triglyceride hydrolysis rate (Lambert et al., 2003). Additionally, FXR also promotes the expression of Phospholipid Transfer Protein (PLTP) to accelerate the removal of lipid substances in HDL (Kast et al., 2001). Overall, the bile acid receptor FXR controls the body's lipid balance by affecting multiple processes, including lipid generation, lipid oxidation metabolism, and lipid transport and uptake, thereby maintaining the body's lipid homeostasis.

### 4.2 Bile acids and G protein-coupled receptor 5 (TGR 5)

A novel bile acid membrane receptor called TGR5 was first discovered by Maruyama et al. in 2002 (Maruyama et al., 2002). TGR5 is a GPCR (a member of the superfamily), also known as GPBAR1 or membrane-type bile acid receptor (M-BAR), and is an important bile acid receptor (Castro et al., 2019). This significant and widely distributed substance is mainly present in various types of cells in different tissues, such as lipid cells, large mononuclear leukocytes, bile duct surface cells, smooth muscle cells within the microvasculature of hepatic sinusoids, and sites of stone formation, among others (Kawamata et al., 2003; Keitel et al., 2009; Reich et al., 2007). It has also been found in some specific organs, such as the insulin-secreting β cells, and its impact at these sites has been identified (Keitel, 2008). Evidence also suggests that this molecule appeared in certain specific brain regions and played specific roles, such as regulating the speed of gastric motility to maintain the normal operation of the digestive system (Poole et al., 2010).

Although all types of bile acids can activate the TGR5 receptor, it exhibits a stronger response to secondary bile acids than to primary bile acids, especially with a specificity for tauroursodeoxycholic acid (TLCA), lithocholic acid (LCA), and deoxycholic acid (DCA; Bunnett, 2014). TGR5 is involved in regulating intestinal motility changes caused by bile acids. The activation of TGR5 can stimulate intestinal contraction; however, excessive TGR5 expression may accelerate intestinal transit, and conversely, the absence of TGR5 may lead to constipation issues (Alemi, 2013). Since TGR5 was expressed in enteric neurons, it played a crucial role in regulating intestinal motility. In vivo experiments have shown that the absence of the TGR5 receptor can lead to disruption of tight junction structures in the intestinal epithelium, thereby affecting intestinal motility (Cipriani et al., 2011; Ichikawa et al., 2012). Furthermore, in terms of fat metabolism, studies have shown that the activation of TGR5 can promote adipocyte differentiation and lipid metabolism, thereby regulating fat accumulation within the body. Additionally, during lipolysis, activation of TGR5 transduces signals to the mammalian target of rapamycin complex I (mTORC1), which was primarily involved in metabolic activities and indirectly participates in fat breakdown, through protein kinase B (AKT; Wang H. et al., 2024). Subsequently, mTORC1 will perform secondary phosphorylation of 4E-binding protein 1 (4E-BP1), thereby regulating the degree of gene translation and ultimately driving the process of fat breakdown (Li et al., 2020). In summary, gut microbes regulate lipid metabolism by affecting adipocyte differentiation and lipid metabolism through bile acid receptor TGR5, thereby maintaining lipid homeostasis.

### 4.3 Bile acids and pregnane X receptor (PXR)

PXR has been identified since 1998 as a member of the superfamily of nuclear receptors (NR). Its presence has been demonstrated in hepatic cells and the intestine in all known mammalian species (Chen et al., 2024; Zollner et al., 2010; Mora et al., 2008). These tissues serve as primary organs responsible for the intake, distribution, metabolism, and clearance of external substances and internal elements. In recent years, with the in-depth study of the intestinal microbiome, researchers have discovered a close interplay between the PXR and the gut microbiota. PXR acted as a nuclear receptor for bile acids and is involved in the biosynthesis, transport, and regulation of bile acids. Additionally, the transcription of CYP7A1 was inhibited by PXR in the liver, which affects bile acid synthesis (Zhou et al., 2023). Further research has also indicated that PXR activation can promote the interactions between PXR and HNF4α and blocked the activation of peroxisome proliferator-activated receptor γ (PPARγ) coactivator 1α by HNF4α, leading to the inhibition of CYP7A1 transcription (Thomas et al., 2022). Furthermore, PXR activation regulated the expression of organic anion transport peptide 2(OATP2), sulfotransferase, and mouse Cyp3A11/human CYP3A4, potentially facilitating bile acid metabolism and transport (Cipriani et al., 2011). This is because OATP2, which was located on the basolateral membrane of hepatic cells and involved in the cellular uptake of bile acids, was induced by PXR and may increase the uptake of bile acids from sinusoidal blood into hepatic cells, where detoxification pathways (such as hydroxylation and sulfation) may occur via Cyp3a11/CYP3A4 and sulfotransferase, respectively (Maglich et al., 2002).

In the intestinal tract, PXR interacted with the gut microbiota. However, the effect of this interaction was further influenced by the composition and function of the gut microbial community. Studies have shown that the activation and deficiency of PXR can affect the diversity and abundance of the gut microbial community (Liu T. et al., 2020). For example, PXR knockout mice had increased gut microbial richness leading to an increase in the pro-inflammatory bacterium *Helicobacter pylori*. PXR ligands, such as bile acids and pregnane X, regulate the metabolic pathways of the gut microbiota, thereby affecting their growth and reproduction (Little et al., 2022). However, when PXR is blocked by statins such as atorvastatin, the intestinal microbial composition of mice is significantly altered, leading to a reduction in the diversity of the host's intestinal microbiota (Benakis et al., 2016). The Firmicutes to Bacteroidetes (F/B) ratio is one of the markers of obesity. In male wild-type mice, a high-fat diet had been shown to upregulate the F/B ratio in a PXR-dependent manner; however, the F/B ratio remained unchanged in female PXR knockout mice after a high-fat diet (Kim et al., 2021). Therefore, PXR may partially promoted the occurrence of high-fat diet-induced obesity through the regulation of changes in the gut microbiome (Xie and Liu, 2021). The activation of PXR can also affect the homeostasis of intestinal microflora and maintain intestinal health (Pushpass et al., 2022). Furthermore, intestinal flora can activate PXR by metabolizing short-chain fatty acids, thereby regulating metabolic processes in the human body, such as cholesterol metabolism and glucose metabolism. However, excessive SCFAs may lead to an imbalance in energy regulation, thereby promoting obesity (Du et al., 2022). The interaction between PXR and the intestinal microbiota is a complex and important research field. In the future, with the in-depth study of the intestinal microbiota and the function of PXR, additional discoveries regarding the mechanisms of their interaction will provide novel ideas and approaches for the prevention and treatment of related diseases.

### 4.4 Bile acids and vitamin D receptor (VDR)

The vitamin D receptor (VDR) is a protein containing six functional domains. Its E domain is mainly used for interacting with molecular partners, and VDR can simultaneously recognize and bind to various ligands, including vitamin D (Zhang et al., 2020). Additionally, it can also exert its activity by recognizing secondary bile acids, especially cholic acid and its derivatives, including lithocholic acid, glycochenodeoxycholic acid, and 7-ketolithocholic acid. However, these secondary bile acids can significantly affect the expression of VDR (Han and Chiang, 2009). The enterohepatic circulation of bile acids may enhance the activity of VDR in the liver and intestines. The concentration of lithocholic acid in the liver and intestines increases, thereby stimulating VDR activity and converting it into more harmless metabolites when cholestasis occurs (Qin and Wang, 2019). Existing studies have confirmed that lithocholic acid can induce the expression of CYP3A4 in the liver through VDR, further promoting the oxidation of bile acids (Cheng et al., 2014), while intestinal VDR deficiency exacerbated liver damage caused by lithocholic acid. However, in transplanted intestine-specific VDR-deficient mice, abnormal overexpression of CYP3A4 led to attenuation of liver damage caused by lithocholic acid (Wang et al., 2022). This demonstrates the association between the expression level of VDR and liver damage in such an environment. Furthermore, some studies have found that in mice with VDR gene knockout, lithocholic acid can trigger VDR activation and increase calcium levels in the blood (Yao et al., 2019).

Some studies indicated that the gut microbiota and its derivatives also influence the function of VDR, such as short-chain fatty acids (propionic acid) and their derivatives (butyrate ester), which were metabolic products of specific microbial communities that enhance the expression of VDR in the gut while mitigating inflammatory responses (Nehring et al., 2007; Hays et al., 2024). On the other hand, the abundance of VDR may potentially influence changes in gut bacteria. A study reported that compared to the gut microbiota of mice with VDR gene deficiency, normal mice had a significant decrease in the population of acid-producing bacteria that were observed in the VDR-deficient mice and a noticeable increase in *Clostridia* and other bacilli (Jin et al., 2015). This further confirms the potential association between VDR and the gut microbiota environment.

## 5 Bile acid metabolism disorder and obesity

In the increasingly fast-paced modern society, obesity is becoming a major concern even in younger populations. As one of the important influencing factors of obesity, disorders in bile acid metabolism have been receiving increasing attention alongside obesity-related indicators, such as body weight, body fat percentage, serum lipid, and related gene expression (Table 2). Moreover, obese individuals often had abnormal bile acid metabolism, which may further promoted the occurrence and development of obesity (Wang and Xue, 2020).

**Table 2:** Effects of bile acid changes on obesity.

**Bile acids**	**Obesity related indicators and gene expression changes**	**References**
Deoxycholic acid↑	Expression of Atgl, Hsl, and Ucp1↑, body fat↓	Tuerhongjiang et al., 2025
Hyodeoxycholic acid↑	Body fat↓, blood fat↓	Kuang et al., 2023; Lu et al., 2020
Ursodesoxycholic acid↑	Weight of HFD mice↓	Jiang et al., 2021
Tauro-ursodesoxycholic acid↑	Body fat↓	Kars et al., 2010
Glycocholic acid↑	Body fat↓	Alves et al., 2019
Hyocholic acid↑	Serum concentrations of TC, TG and LDL-C↓	Yang et al., 2022
Taurocholic acid↑	Hepatic fat content↑, TG↑	Xu et al., 2022
Cholic acid/chenodeoxycholic acid↓	Body weight and body fat↓	Yang et al., 2023
Lithocholic acid↑	AMPK↑, fat content↓, body fat↓	Qu et al., 2025; Wang et al., 2019
Taurochenodeoxycholic acid↑	Fat content↓	Lu et al., 2025

Bile acid is a major influencing factor in the digestion and absorption of fats (Weida et al., 2020). Bile acid metabolism disorders mainly manifests as reduced bile acid synthesis and obstructed excretion in obese populations (Xie et al., 2016). This may be the result of the combined effects of obesity-induced insulin resistance, lipid metabolism disorder, and impaired liver function, which led to increased fat accumulation, thereby triggering obesity (Buzzetti et al., 2016; Gonzalez et al., 2016). Watanabe et al. fed bile acid (CA) to obese mice induced by high-fat diet and observed that bile acid metabolism disorders affected the balance of gut microorganisms, further impacting the occurrence of obesity and metabolic diseases (Watanabe et al., 2006). Studies have also indicated that bile acids affect the composition of the gut microbiota through their partial antibacterial properties or involvement in the immune function of the intestinal mucosa, thereby influencing the occurrence of obesity (Cui and Gao, 2022). Therefore, investigating and understanding the link between bile acid metabolism imbalance and obesity plays an extremely important role in the prevention and treatment of obesity and related diseases. Future research should further explore disorders in bile acid metabolism and how to prevent and treat obesity and related diseases by regulating bile acid metabolism. Additionally, approaches for optimizing bile acid metabolism can be explored in obese populations to reduce the risk of obesity and related diseases while also maintaining healthy lifestyle habits such as a balanced diet and moderate exercise.

## 6 The bile acid pathway alleviates lipid metabolism disorders through gut microbiota

Bile acids are a class of substances synthesized by cholesterol in the liver and play a key role in fat digestion and absorption. In recent years, studies have shown that the bile acid pathway effectively alleviated lipid metabolism disorders under the influence of the gut microbiota. For example, inulin promoted the excretion of bile acids and reduced the reabsorption of bile acids by increasing the alpha diversity of gut microbiota (Wang et al., 2022). This led to a reduction in the serum triglyceride (TG) and low-density lipoprotein (LDL) levels, thereby improving lipid metabolism in animals (Zhang et al., 2025). Studies had shown that feeding tetrahydropalmatine altered the structure of intestinal flora, effectively regulated bile acid metabolism, and improved abnormal lipid metabolism in ApoE gene knockout mice (Gao, 2024). In-depth studies have also reported that peanut red proanthocyanidins significantly increased the level of *Akkermansia muciniphila* and *Parabacteroides distasonis* in the intestine of obese type II diabetic mice (Masumoto et al., 2016; Roopchand et al., 2015). It had also been shown that *Akkermansia* is negatively correlated with obesity and diabetes, as the consumption of *Akkermansia* significantly reduces body weight while improving lipid metabolism abnormalities and blood glucose levels (Everard et al., 2013). Additionally, a gavage of *Parabacteroides distasonis* increases the levels of LCA, UDCA, and succinic acid in the gut; LCA and UDCA reduce blood lipids by activating the FXR pathway and repair the intestinal barrier, while succinic acid reduces hyperglycemia in ob/ob mice by activating intestinal gluconeogenesis, thereby improving lipid metabolism disorders (Wang et al., 2019). Thus, some plant extracts and microecological preparations effectively improve lipid metabolism disorders through the intestinal microbiota-bile acid pathway.

## 7 Conclusion and outlook

Bile acids, as crucial messengers between the gut microbiota and the host, play an important role in lipid metabolism. This review mainly explores the synthesis, metabolism, and regulatory role of bile acids in lipid metabolism and further discusses the key roles of bile acid receptors in bile acid metabolism and lipid metabolism. FXR, PXR, VDR, and TGR5, as important bile acid receptors, participate in the synthesis, transportation, metabolism, and excretion of bile acids by regulating the expression of related genes, maintaining the balance of bile acids in the body, and stabilizing lipid metabolism. Additionally, bile acids interact with the gut microbiota and the host, influencing lipid metabolism and overall health. This study comprehensively explores the interactive relationship between intestinal bile acids and microorganisms from the perspective of nutrition science, as well as the regulatory mechanism of the interaction on lipid metabolism. Furthermore, we provided some nutritional strategies that alleviate lipid metabolism disorders through the intestinal microbiota-bile acid pathway.

Although some progress has been made in the study of bile acids regulating host lipid metabolism through intestinal flora, there are still many unresolved questions. First, it is necessary to deeply understand the interaction mechanism between intestinal microbiota and bile acid metabolism and clarify the specific contribution and regulatory mechanism of various microbiota to bile acid metabolism. Second, beneficial gut microbiota need to be identified, while also exploring improvements in the host's lipid metabolism through the regulation of the gut microbiota community structure. Furthermore, the mechanisms of interaction between the gut microbiota and the host require further research.
